# Highly Sensitive and Quantitative Diagnosis of SARS-CoV-2 Using a Gold/Platinum Particle-Based Lateral Flow Assay and a Desktop Scanning Electron Microscope

**DOI:** 10.3390/biomedicines10020447

**Published:** 2022-02-15

**Authors:** Hideya Kawasaki, Hiromi Suzuki, Kazuki Furuhashi, Keita Yamashita, Jinko Ishikawa, Osanori Nagura, Masato Maekawa, Takafumi Miwa, Takumi Tandou, Takahiko Hariyama

**Affiliations:** 1 Institute for NanoSuit Research, Preeminent Medical Photonics Education & Research Center, Hamamatsu University School of Medicine, 1-20-1 Handayama, Higashi-ku, Hamamatsu 431-3192, Japan; suzuhiro@hama-med.ac.jp (H.S.); hariyama@hama-med.ac.jp (T.H.); 2 Department of Laboratory Medicine, Hamamatsu University School of Medicine, 1-20-1 Handayama, Higashi-ku, Hamamatsu 431-3192, Japan; k.furu@hama-med.ac.jp (K.F.); keitay@hama-med.ac.jp (K.Y.); jin0406@hama-med.ac.jp (J.I.); rikyo@hama-med.ac.jp (O.N.); mmaekawa@hama-med.ac.jp (M.M.); 3 Research & Development Group, Hitachi Ltd., 1-280, Higashi-Koigakubo, Kokubunji-shi, Tokyo 185-8601, Japan; takafumi.miwa.gn@hitachi.com; 4 Social Solution Department, Hitachi Social Information Services Ltd., Omori Bellport D 17F, 6-26-3, Minamioi, Shinagawa-ku, Tokyo 140-0013, Japan; takumi.tando.ee@hitachi.com; 5 NanoSuit Inc., 1-20-1 Handayama, Higashi-ku, Hamamatsu 431-3192, Japan

**Keywords:** desktop scanning electron microscopy, SARS-CoV-2, lateral flow assay, NanoSuit, COVID-19

## Abstract

The gold standard test for identifying SARS-CoV-2, the causative agent of COVID-19, is polymerase chain reaction (PCR). Despite their limited sensitivity, SARS-CoV-2 antigen rapid diagnostic tests are vital tools in the fight against viral spread. Owing to its simplicity and low cost, the lateral flow assay (LFA) is the most extensively used point-of-care diagnostic test. Here, we report a newly designed LFA-NanoSuit method (LNSM) that works in conjunction with desktop scanning electron microscopy (SEM) to detect SARS-CoV-2. LNSM requires no standard SEM treatment, avoids cellulose and residual buffer deformation, and enables the capture of high-resolution images of antibody-labeled gold/platinum particles reacting with SARS-CoV-2 antigens. To assess its applicability, we compared clinical SARS-CoV-2 samples via visual detection of LFA, LSNM detection of LFA, and real-time reverse transcription-PCR (qRT-PCR). Compared to qRT-PCR, LNSM showed 86.7% sensitivity (26/30; 95% confidence interval (CI): 69.28–96.24%) and 93.3% specificity (14/15; 95% CI: 68.05–99.83%) for SARS-CoV-2. In samples with a relatively low SARS-CoV-2 RNA copy number (30 < Ct ≤ 40), the sensitivity of LNSM was greater (73.3%) than that of visual detection (0%). A simple, sensitive, and quantitative LNSM can be used to diagnose SARS-CoV-2.

## 1. Introduction

Severe acute respiratory syndrome coronavirus 2 (SARS-CoV-2) is a serious threat to public health and the global economy. The World Health Organization (WHO) designated the coronavirus disease 2019 (COVID-19) outbreak as a pandemic in March 2020. [1]. To date, according to the WHO, the global cumulative number of novel coronavirus infection cases exceeds 300 million [2]. Rapid identification and isolation of patients diagnosed with SARS-CoV-2 infection is important to prevent nosocomial transmission. Real-time reverse transcription–polymerase chain reaction (qRT-PCR) of samples from nasopharyngeal swabs, sputum, various lower respiratory tract secretions, and saliva of patients is the most widely used diagnostic method for COVID-19 diagnosis [3]. Nevertheless, its adoption has been hindered by factors such as high cost, limited scalability, personnel training, and quality control measures [4]. Using the lateral flow assay (LFA) to diagnose SARS-CoV-2 infection offers a potential point-of-care option that may be obtained in near-infinite quantities and performed at the bedside in 10 min. However, its use in clinical settings has been under debate owing to its low sensitivity compared to qRT-PCR [5], and it requires an experienced operator, which influences the test performance [4].

We previously described a new LFA-NanoSuit method (LNSM) that works in conjunction with desktop scanning electron microscopy (SEM). It combines LFA with the NanoSuit method to prevent deformation of immunochromatography substrates, such as cellulose and residual liquid, resulting in fuzzy particle images when observed via SEM [6]. The NanoSuit method is a simple procedure for SEM examination of multicellular organisms, in which the sample is encased in a thin, vacuum-sealed casing that can be used for medicinal applications [7,8,9]. Moreover, unlike traditional SEM, the LNSM enables easy focusing and gathering of high-resolution images without the need for additional conductive treatment. For influenza A, the detection ability of the LNSM is comparable to that of qRT-PCR [6]. Here, we compared the detection capacity of various diagnostic methods for SARS-CoV-2 using laboratory and clinical samples from patients with SARS-CoV-2 infection, including the nasopharynx, nasal cavity, and saliva. Specifically, we analyzed the specificity and sensitivity of LFA compared with those of qRT-PCR via visual detection and LNSM.

## 2. Materials and Methods

### 2.1. Lateral Flow Strip Preparation

An ImunoAce^®®^ SARS-CoV-2 kit was used to detect the SARS-CoV-2 nucleocapsid protein (NP) antigen (TAUNS Laboratories, Shizuoka, Japan). Anti-mouse IgG antibody and anti-SARS-CoV-2 NP antibody were immobilized on chromatographic paper, whereas the other anti-SARS-CoV-2 NP antibody was tagged with colloidal gold/platinum (Au/Pt) and infiltrated into the sample pad. The sample pad was then attached to the end of the membrane. A positive line was detectable when Au/Pt nanoparticles (100–300 nm) were captured.

### 2.2. Preparation of Clinical Samples

A total of 88 clinical samples from 45 suspected COVID-19 patients (Table 1) were examined using the ImunoAce^®®^ SARS-CoV-2 kit and visual detection at Hamamatsu Medical Center and Hamamatsu University Hospital. Nasopharyngeal, nasal, and saliva samples were collected from the same individuals at Hamamatsu Medical Center (*n* = 65 from 22 patients) (Table 1: patient Nos. 1–22). Nasopharyngeal samples were obtained from 23 patients at Hamamatsu University Hospital (Table 1: patient Nos. 23–45). LFA and qRT-PCR samples were obtained separately for the same patient. The LFA tests were performed immediately after collecting the samples. The samples for qRT-PCR were stored in a transport medium at 4 °C in a refrigerator. The LFA kits were stored in a biosafety container at room temperature (20–25 °C). The study was conducted in accordance with the Declaration of Helsinki and was approved by the Ethics Committee of Hamamatsu University School of Medicine (No. 19–134 (14 July 2019), No. 20–250 (12 November 2020)) and Hamamatsu Medical Center (No. 2021-074) (31 August 2021) for studies involving humans.

### 2.3. Standard Solution of SARS-CoV-2 Nucleocapsid Protein Antigen

Preparation Stock solution (1 mg/mL) of the human recombinant NP of SARS-CoV-2 (HEK293) and the His-tag C-terminus (Diaclone SAS, Besançon Cedex, France) was prepared. First, a series of working solutions (0, 0.1, 1, 10, 10^2^, 10^3^, 10^4^, and 10^5^ pg/mL) was prepared by diluting the stock solution with different volumes of buffer. Subsequently, the diluted solution buffer (120 μL) was slowly applied to the sample region of the test strip.

### 2.4. Method of Visual Detection for the Test Strip

Three drops (80–120 μL) of clinical sample solution and 120 μL of laboratory sample solution were gradually applied to the test strip sample area to determine the diagnostic cut-off point. Results obtained within 10 min were deemed valid and those obtained after 15 min were deemed invalid. Two investigators read the test line and classified it as “positive”, “negative”, or “positive to undetermined”. The investigators were blinded to the results of each type of test when samples were obtained from the same individual.

### 2.5. Densitometry Detection Method for the Test Strip

The intensity of the test lines on the test strips was determined using an Immunochromato-Reader C10066–10 (Hamamatsu Photonics, Hamamatsu, Japan). The values are expressed in milli-absorbance units (mABS).

### 2.6. Single-Step qRT-PCR for SARS-CoV-2

SARS-CoV-2 qRT-PCR was performed using the orf1ab set, which included a forward primer (orf1ab-13215-F: 5′-CCGGAAGCCAATGGATCA-3′), reverse primer (orf1ab-13257-R: 5′-GCAACGGCAGTACAGACAACA-3′), and probe (orf1ab-13238-P: FAM-ATCCTTTGGTGGCATC-MGB) (Sysmex Corporation, Kobe, Japan). The Quantstudio^®®^ 5 real time PCR system (Thermo Fisher Scientific, Tokyo, Japan) was used to perform qRT-PCR. According to the protocol, a cycle threshold (Ct) value of ≤40 was considered as a positive result [10].

### 2.7. SEM Image Acquisition

As previously described [6], the LFA kit’s cellulose pad was coated with a modified NanoSuit^®®^ solution with Tween-20-based components (Nisshin EM Co., Ltd., Tokyo, Japan), mounted on the wide stage of the specimen holder, and then placed under a desktop scanning electron microscope (TM4000Plus, Hitachi High-Technologies, Tokyo, Japan). Backscattered electron detectors, operating at 10 or 15 kV and 30 Pa, were used to capture the images [6].

### 2.8. Particle Counting

Images were processed and particles were counted according to a previously reported methodology [11]. The particles were manually counted in fields with less than 50 particles per field. In all other fields, the particles were counted using ImageJ/Fiji software (National Institutes of Health). To differentiate particles, ImageJ/Fiji uses sophisticated particle analysis techniques.

### 2.9. SEM Diagnosis and Statistics

Statistical analysis using Student’s *t*-test was carried out using Microsoft Excel version 16.57 (Microsoft, Redmond, WA, USA). A receiver operating characteristic (ROC) curve was created and analyzed using software from International Business Machines Corporation’s statistical package for social sciences (IBM Corp., Armonk, NY, USA). The ROC curve was used to compare the accuracy of the diagnostic test with that of a reference/gold standard test. MedCalc (MedCalc software, Ostened, Belgium) was used to conduct the statistical analysis of the sensitivity and specificity of the assay at a 95% confidence interval (CI). Limit of detection (LOD) was defined as the mean blank signal plus 3.3-times the standard deviation (SD) of the blank (LOD = mean_blank_ + 3.3 × SD _blank_) [12].

The average number of particles from test line (TL) and background area (BA) were compared by counting six fields of view at 1200× magnification. According to the observational data and statistical analysis, if there was more than one particle on an average in a single visual field (1200×), and the average ratio of TL/BA was >2, the result was considered positive. The approximation line, correlation coefficient, and null hypothesis were calculated using Excel (Microsoft).

## 3. Results

### 3.1. Lateral Flow Principle for Desktop SEM Detection

**Figure 1:**
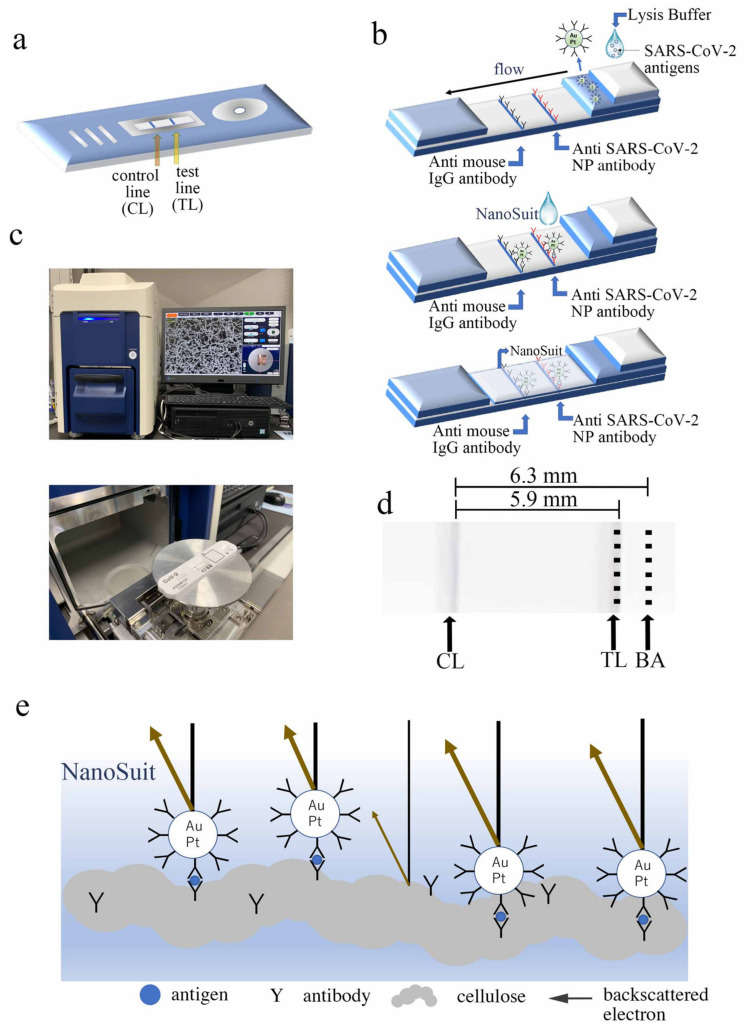
NanoSuit method was used to observe the lateral flow assay (LFA) results: (**a**) Test line (TL) and control line (CL) on the LFA test strip. (**b**) Schematic of the Au/Pt–Ab conjugate-linked fast LFA kit. At the TL, the immune complex reacts with anti-SARS-CoV-2 nucleocapsid protein (NP), whereas at the CL, it reacts with anti-mouse IgG antibody (top, middle). After NanoSuit treatment, a NanoSuit layer is produced (bottom). (**c**) Desktop scanning electron microscope (miniscope TM4000plus; top) used for imaging. Placement of the test strip in the scanning electron microscope chamber (bottom). (**d**) Establishment of observation points. In the TL and background area (BA) sections, six fields were chosen for analysis. (**e**) Schematic of backscattering electrons from Au/Pt particles and the cellulose surface coated with the NanoSuit layer.

Diagnostic kits were prepared to test the sensitivity and specificity of SARS-CoV-2 diagnosis (Figure 1a). A black line formed on the test strip when the Au/Pt particles combined with SARS-CoV-2 antigen in clinical samples, which were placed on the sample pad in lysis buffer, indicating the presence of the desired antigen in the sample (Figure 1b). NanoSuit solution was then introduced upstream of the test line (Figure 1b), causing a thin layer of NanoSuit liquid to develop (Figure 1b,e). The LFA kit was installed on a sample stage of a desktop scanning electron microscope to be as close as possible to the camera (Figure 1c). At set distances from the control line boundary, the TL and BA observation stations were established. The observation areas were defined as the areas with the highest number of TL signals. The TL observation line was 5.9 mm from the right edge of the control line and the BA observation area was 6.3 mm from the right edge of the control line (Figure 1d). The average number of particles from TL and BA were compared by counting six fields of view at 1200× magnification. The electron beam penetrated the NanoSuit layer, as shown in Figure 1e, and released considerably more backscattered electrons from the Au/Pt particles than from the cellulose surface.

### 3.2. Observation of Au/Pt Particles following NanoSuit Treatment

**Figure 2:**
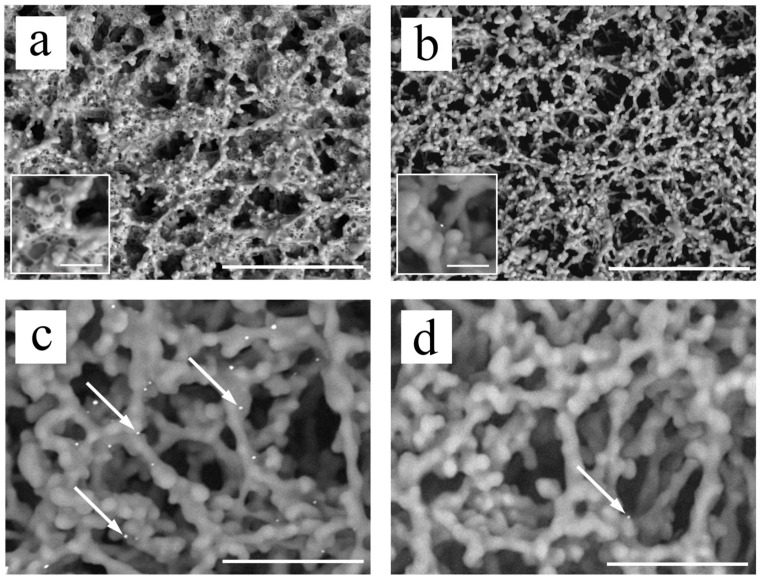
SEM visualization of Au/Pt particles on cellulose: (**a**,**b**) Representative SEM images of cellulose before (**a**) and after (**b**) NanoSuit treatment. Scale bar: 40 μm. The inset is the magnified image. Scale bar: 4 μm. (**c**,**d**) SEM images of the “positive visual detection” test line and background area, respectively, of the LFA kit. Representative Au/Pt particles are indicated with white arrows. Scale bar: 10 μm.

In the absence of NanoSuit treatment, cellulose and leftover lysis buffer deformation was observed as a result of high electron beam energy (Figure 2a). Meanwhile, the cellulose membrane showed little to no swelling and no residual lysis buffer after being treated with NanoSuit (Figure 2b). The multiple Au/Pt particles at the TL (arrows) were visualized (Figure 2c), and only a few were found in the BA of the cellulose membrane (Figure 2d).

### 3.3. Determination of the Diagnostic Cut-Off Point

**Figure 3:**
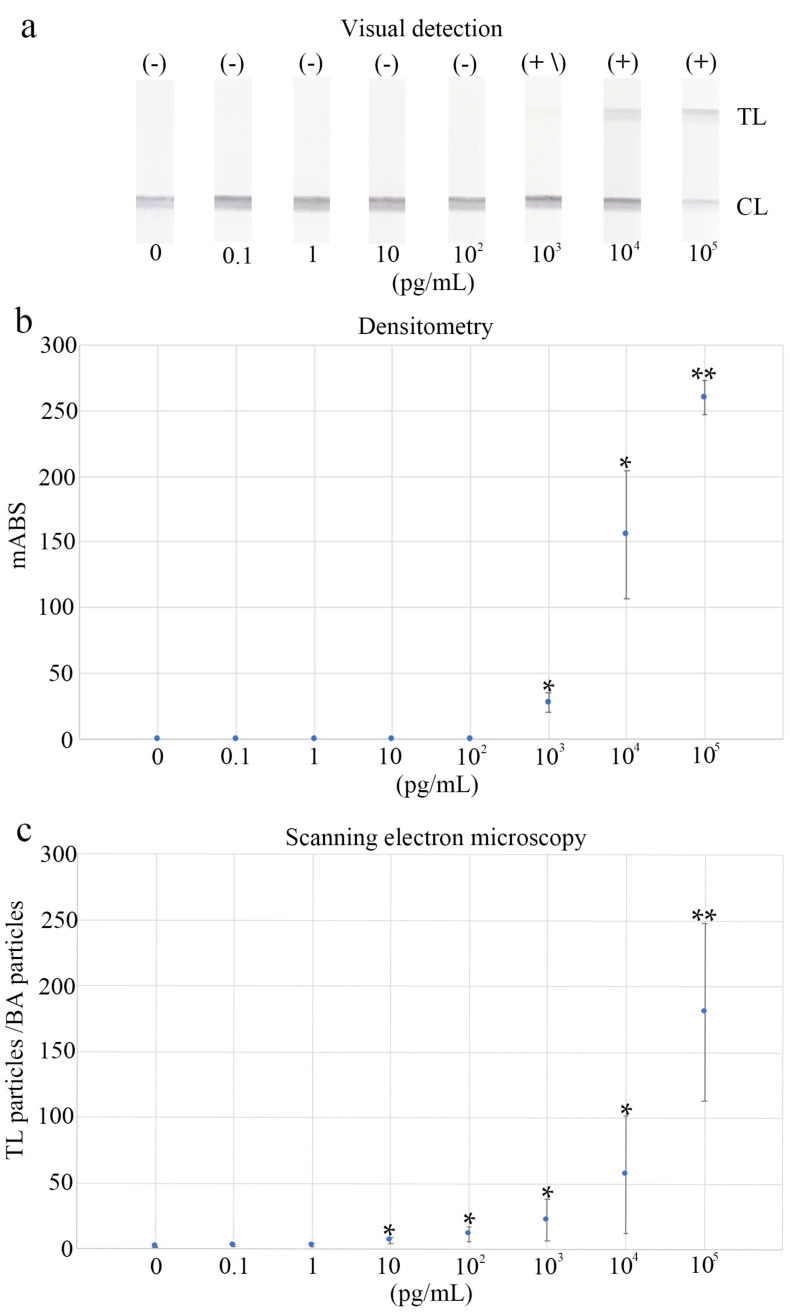
Visual observation, densitometry, and scanning electron microscopy (SEM) were used to compare the performance of the lateral flow assay (LFA): (**a**) Visual detection of a diluted series of SARS-CoV-2 nucleocapsid protein samples. (+) represents positive, (+\) represents positive to undetermined, (-) represents negative. (**b**) Densitometry and (**c**) SEM detection using the same dilution series of SARS-CoV-2 nucleocapsid protein samples as in (**a**). * *p* < 0.05, ** *p* < 0.01. TL, test line; CL, control line; mABS, milli-absorbance units; BA, background area.

Human recombinant NP of SARS-CoV-2 at a dose of 10^4^ pg/mL was categorized as “positive” with visual detection, whereas the human recombinant NP of SARS-CoV-2 at a dose 10^3^ pg/mL caused a low signal and was classified as “positive to indeterminate” (Figure 3a). Identical LFA samples were used for diagnosis using densitometry and SEM. The LOD was 0 mABS for densitometry analysis. The result was considered affirmative when the sample value surpassed the LOD. Regardless of lot-to-lot variance, doses > 10^3^ pg/mL of the human recombinant NP of SARS-CoV-2 were significantly different from the LOD (*p* < 0.05), suggesting a sensitivity approximately 10-fold greater than that of the visual diagnosis (Figure 3b; Table S1). For the SEM study, the LOD was 2.023, defined by the signal-to-background ratio (TL/BA ratio). The 10 pg/mL dose of the human recombinant NP of SARS-CoV-2 was significantly different from that of the LOD (*p* < 0.05), showing a 100- to 1000-fold increase in sensitivity over visual diagnosis, despite lot-to-lot variation (Figure 3c; Table S2).

### 3.4. Visual, Scanning Electron Microscopy, and qRT-PCR Methods Compared in Terms of Clinical Diagnostic Sensitivity

**Figure 4:**
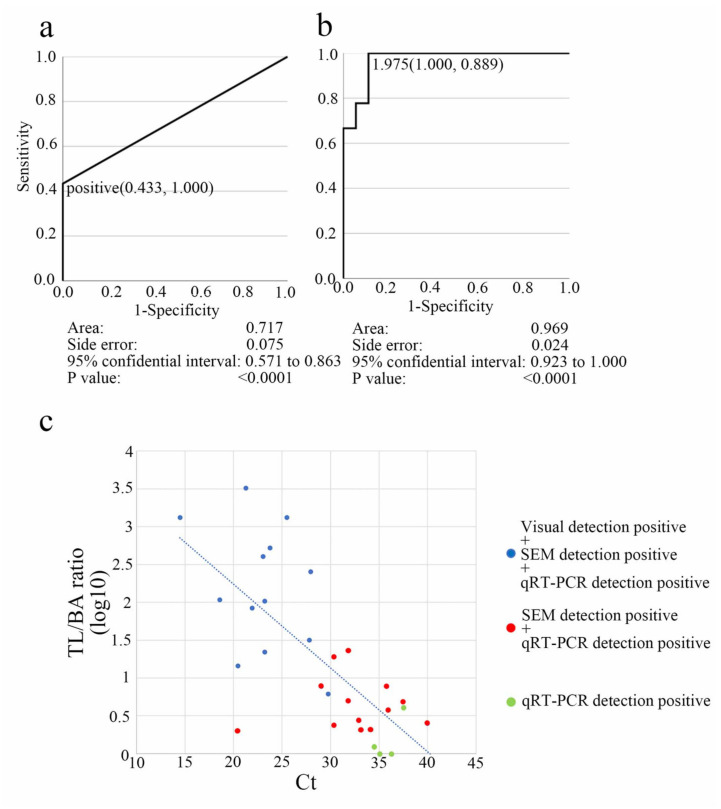
Comparison of lateral flow assay (LFA) detection methods for SARS-CoV-2 using clinical samples: (**a**) The accuracy of visual diagnosis was assessed by testing SARS-CoV-2-negative and -positive patients using qRT-PCR and the antigen solutions from the same patients, and then calculated the area under the curve (AUC) for the receiver operating characteristic (ROC) curve. (**b**) Detection via scanning electron microscopy (SEM) using the test line (TL)/background area (BA) ratio. A TL/BA ratio of 1.975 was chosen as the threshold cut-off value based on the ROC curve study to differentiate SARS-CoV-2 infection. (**c**) TL/BA ratio (log10) and cycle threshold (Ct) scatter plot. Triple-positive results (“visual detection-positive”, “SEM detection-positive”, and “qRT-PCR detection-positive”) are represented with blue dots, double-positive results (“SEM detection-positive” and “qRT-PCR detection-positive”) are represented with red dots, and single-positive results (“qRT-PCR detection-positive”) are represented with green dots. The approximate slope is indicated by the blue dotted line.

For the differential diagnosis of SARS-CoV-2, clinical nasopharyngeal swab samples (n = 45) were examined using LFA and qRT-PCR. We detected 30 positive and 15 negative SARS-CoV-2 cases using qRT-PCR. The sensitivity and specificity of visual and SEM detection of LFA were then compared with those of qRT-PCR. The ROC curve was used to estimate the diagnostic cut-off value. The area under the curve (AUC) value for visual diagnosis was 0.717 (95% CI: 57.1–86.3%; *p* < 0.0001), with a specificity of 0.433 and a sensitivity of 1.000 (Figure 4a; Table S3). The TL/BA ratio of SEM detection was evaluated using the ROC curve. With a specificity of 1.000 and a sensitivity of 0.899, the AUC value for the SEM-based TL/BA ratio diagnosis was 0.969 (95% CI: 92.3–100%; *p* < 0.0001), with a cut-off value of 1.975 (Figure 4b). This is comparable with previous cut-off values (2.023) for the dilution assay (Figure 3c; Table S2). According to the observational data and statistical analysis, if there was more than one particle on an average in a single visual field (1200×), and the average ratio of TL/BA was >2, the result was considered positive. In Figure 4c, scatter plots depict the quantitative association between the TL/BA ratio (log_10_) and Ct values (Table S3). Because the correlation coefficient between Ct and particle counts/field was −0.427 and statistically significant (*p* = 0.00866), the null hypothesis was rejected.

SEM detection for LFA of SARS-CoV-2 exhibited 86.7% sensitivity (26/30; 95% CI: 69.28–96.24%) and 93.3% specificity (14/15; 95% CI: 68.05–99.83%; Table 2 and Table S3). The results were nearly identical (kappa = 0.889) to those obtained via qRT-PCR (10.0 < Ct ≤ 40.0; Table 3 and Table S3). Visual detection had a clinical sensitivity of 43.3% (13/30; 95 percent CI: 25.43–62.57%), a clinical specificity of 100% (95% CI: 78.2–100%), and a close match (kappa = 0.622) with qRT-PCR data (10.0 < Ct ≤ 40.0; Table 3, and Table S3). As confirmed using qRT-PCR, SEM-based identification was more sensitive (73.3%) than visual detection (0%), particularly in samples with a lower SARS-CoV-2 RNA copy number (30 < Ct ≤ 40; Table 2 and Table S3).

**Figure 5:**
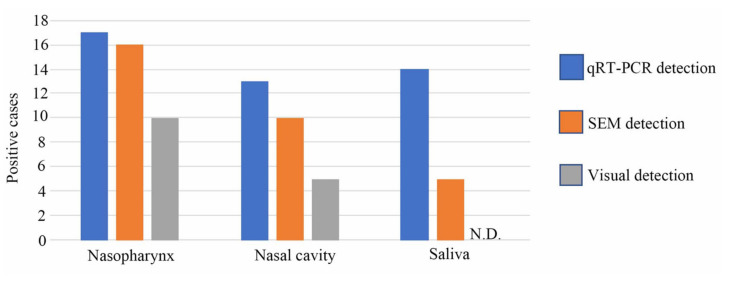
Comparison of detection methods among different sample types. Samples of the nasopharynx, nasal cavity, and saliva collected from the same person were compared using visual, scanning electron microscopy, and qRT-PCR detection methods. N.D. represents “not detected”.

Samples from the nasopharynx, nasal cavity, and saliva of the same person were compared using visual, SEM, and qRT-PCR detection methods. SEM detection via LFA showed 94.1%, 76.9%, and 35.7% sensitivity for the nasopharynx, nasal cavity, and saliva samples, respectively, compared with that of qRT-PCR. However, visual detection via LFA showed 58.8%, 38.5%, and 0% sensitivity in the nasopharynx, nasal cavity, and saliva samples, respectively, compared with that of qRT-PCR (Figure 5 and Table S3). Analysis of samples collected from the nasopharynx is the most sensitive among all samples tested using LNSM.

## 4. Discussion

The LFA is an easy, low cost, rapid, and qualitative diagnostic tool. The LFA’s visual sensitivity varies depending on the observers and LFA’s reaction time (with shorter reaction time showing lower sensitivity). However, a visual diagnosis of LFA is the most extensively used point-of-care diagnostic test despite its qualitative characteristics. In our study, scatter plots demonstrated the inverse quantitative relationship between TL/BA ratio (log10) and Ct (Figure 4c). LNSM can add a quantitative factor to conventional LFA kits, providing high sensitivity.

In a previous study, ultimate sensitivity was demonstrated for the Au/Pt-based LFA in detecting influenza virus A using LNSM and considering antigen–antibody affinity and lot-to-lot differences [6,13]. LNSM should have the highest LFA sensitivity as it involves direct monitoring of conventional metal particles. We applied this technology to diagnose COVID-19. In this study, the sensitivity of LNSM, which was tested using SARS-CoV-2 NP antigens, was approximately 100–1000 times higher than that of visual detection (Figure 3; Table S2). Importantly, using clinical samples, LNSM showed a sensitive detection level (73.3%) that was higher than that of visual detection (0%), particularly in samples with a relatively low SARS-CoV-2 RNA copy number (30 < Ct ≤ 40) (Table 2 and Table S3). As a result, our study demonstrates the ultimate sensitivity of LFA employing Au/Pt for SARS-CoV-2 detection. In some cases, outliers and false positive/negative cases were observed; this may have been because the samples were collected separately for LFA and qRT-PCR, despite being collected from the same site from the same person.

The link between Ct value and infectivity is debatable. Patients with Ct values of >33 to 34 do not spread the infection and can be discharged from the hospital, according to a link between successful virus isolation in cell culture and the qRT-PCR Ct value [14]. Bullard et al. [15] found that SARS-CoV-2 infectivity in Vero cells was detected only when the qRT-PCR Ct was < 24. Patients with a Ct > 24 and symptoms that last longer than 8 days may have low infectious potential. Another research group found that 5 of 60 patients with a Ct > 35 transmitted the virus. Furthermore, all five samples were obtained from symptomatic people with no evidence of severe illness. There is an estimated 8.3% risk of viral recovery from samples with a Ct > 35 (95% CI: 2.8–18.4%) [16]. In cell culture, virus growth is efficient in samples with Ct values between 10 and 20 (76.7% positive isolation rate). Still, virus growth decreased to 24.1% in samples with Ct values between 20 and 30, and to 2.9% in samples with values between 30 and 40 [17]. When the Ct value of PCR tests was compared to the sensitivity of various rapid antigen test results of different sample types (e.g., mouthwash, saliva, nasopharyngeal swab, and sputum) from COVID-19 patients, a Ct > 30 indicated that the isolation culture of the virus could not be obtained [18]. Thus, according to the studies mentioned above, individuals with a Ct > 35 have a low risk of transmitting SARS-CoV-2.

LFA is suitable for detecting COVID-19 in individuals who are shedding a considerable amount of SARS-CoV-2; thus, the technique may be beneficial in identifying patients who are at a high risk of transmitting the virus. However, several samples from where the virus was recovered tested negative using LFA, implying that the method may not be able to diagnose all individuals who are shedding infectious SARS-CoV-2 [19]. On the contrary, regardless of their clinical status, 50% of the people who test positive for SARS-CoV-2 through qRT-PCR appear to be in the noninfectious phases of the disease, as shown by low viral loads being in a range from which live viruses are rarely isolated. Only 2% of people carry 90% of the virus that circulates in communities, thus serving as viral “supercarriers” and likely also as “super spreaders” [19]. Frequent tests, such as antigen tests, which are slightly less sensitive but simple, fast, and inexpensive, are more likely to identify individuals at a high risk of infection before and during viral load peaks [20]. The gold standard clinical PCR test fails to meet numerous requirements when used in a surveillance routine. Following collection, PCR samples are often transported to a centralized laboratory staffed by experts, increasing the expense, decreasing the frequency, and potentially delaying results by one or more days. Highly sensitive LNSM may be beneficial in efficiently identifying the true virus-shedding patients and in reducing the number of tests required for surveillance testing.

Saliva collection is a non-invasive and self-collection method that reduces the strain on health care providers, risk of infection, pain experienced during testing, and physical expenditures associated with personal protective equipment. As a result, saliva collection is particularly advantageous when collecting a large number of samples in a short period, such as when screening for asymptomatic individuals. Our results indicate that the LFA of saliva had decreased sensitivity when visual and SEM detections were used (Figure 5). Our finding is consistent with that of a previous study, which reported that the pooled sensitivity of rapid antigen diagnostic tests against SARS-CoV-2 changes across collection sites [21]. Therefore, the LFA kit manufacturers must work on developing a saliva-based LFA for SARS-CoV-2. Combining the LFA for a SARS-CoV-2 saliva kit with LNSM may provide the maximum sensitivity for screening asymptomatic individuals, efficiently. LFA requires 10 min of antigen–antibody reaction time, and the LNSM analysis requires about 10 min for each LFA sample, including vacuum and scanning time. Real-time PCR takes 2–4 h, including sample preprocessing and reaction. The time required for LNSM is considerably shorter than that for real-time PCR. At the present technical level, both real-time PCR and LNSM require trained specialists. Although the current desktop SEM was developed as a user-friendly interface, to increase the clinical use of SEM, future research should also focus on developing desktop scanning electron microscopes with a quick vacuum, high scanning speed (preferably less than 3 min for each test), autonomous staging control, and particle counting systems based on artificial intelligence. Similarly, enhancing the sensitivity and specificity of the LFA kit will significantly boost the SEM detection approach. Reducing the cost is an important aspect to spread this technology. Although it is difficult to accurately compare the cost because of the differences in equipment and kits, the cost of the desktop SEM system is approximately the same as the real-time PCR machine in Hamamatsu University Hospital. The LFA kit per test is approximately 37% cheaper than a PCR per test in Hamamatsu University Hospital. Currently, the cost of the LNSM is comparable with that of the standard real-time PCR test. We are now trying to develop a convenient and cheaper LFA kit and a dedicated desktop SEM system for this method. Therefore, we may have a suitable measuring system in the near future.

## 5. Conclusions

According to our findings, the LNSM, including the recently developed SARS-CoV-2 LFA, can be effectively employed for the automated quantitative measurement of SARS-CoV-2 antigens. Overall, the LNSM can help advance the use of LFAs for the rapid diagnosis of new infections and may be applicable to other infectious diseases.

## Figures and Tables

**Table 1:** Clinical samples from suspected COVID-19 patients.

Patient No.	Age	Sex (M/F)	Collection Day after Onset	Diagnosis	Symptom(s)	Alive/Dead
1	46	F	8	COVID-19	fever, muscle pain, headache	alive
2	58	M	37	COVID-19	fever, sore throat, cough	alive
3	55	F	13	COVID-19	sore throat	alive
4	38	M	11	COVID-19	fever	alive
5	45	F	-	Non-COVID-19	fever	alive
6	51	M	8	COVID-19	fever	alive
7	40	M	7	COVID-19	sore throat	alive
8	18	F	5	COVID-19	suffocation	alive
9	65	M	24	COVID-19	no symptoms	alive
10	51	M	13	COVID-19	cough, chills, nasal discharge, sputum, malaise, smell disorder, dysgeusia	alive
11	48	M	11	COVID-19	fever	alive
12	46	M	11	COVID-19	sore throat	alive
13	47	M	-	Non-COVID-19	no symptoms	alive
14	43	F	-	Non-COVID-19	sore throat, diarrhea	alive
15	20	M	9	COVID-19	fever, nasal discharge, sputum, smell disorder, dysgeusia	alive
16	37	F	-	Non-COVID-19	cough, sputum	alive
17	65	M	5	COVID-19	fever	alive
18	60	F	-	Non-COVID-19	no symptoms	alive
19	57	M	7	COVID-19	fever, headache	alive
20	25	M	8	COVID-19	fever, cough	alive
21	61	F	9	COVID-19	sore throat, joint pain, headache	alive
22	76	M	8	COVID-19	fever, sore throat	alive
23	61	M	-	Non-COVID-19	fever	alive
24	56	M	-	Non-COVID-19	fever	alive
25	90	F	43	COVID-19	fever	alive
26	75	F	-	Non-COVID-19	cough, fever	alive
27	34	M	-	Non-COVID-19	fever	alive
28	69	M	-	Non-COVID-19	fever	alive
29	55	M	-	Non-COVID-19	vomiting, fever	alive
30	52	F	-	Non-COVID-19	fever	alive
31	75	M	1	COVID-19	dyspnea	dead
32	74	M	10	COVID-19	fever	alive
33	28	M	9	COVID-19	cough, fever, sore throat	alive
34	45	M	7	COVID-19	headache, loose stool	alive
35	77	M	2	COVID-19	fever	alive
36	82	F	12	COVID-19	dyspnea	dead
37	66	M	14	COVID-19	dyspnea	alive
38	66	M	33	COVID-19	dyspnea	alive
39	66	M	37	COVID-19	dyspnea	alive
40	47	M	-	Non-COVID-19	malaise	alive
41	56	M	-	Non-COVID-19	fever, dyspnea	alive
42	43	M	19	COVID-19	fever	alive
43	48	M	-	Non-COVID-19	fever	alive
44	70	M	13	COVID-19	fever, diarrhea	alive
45	72	M	20	COVID-19	Fever	alive

**Table 2:** Clinical diagnosis performance of the SEM detection method.

Clinical Diagnosis Performance of SEM Detection Method					
SARS-CoV-2 rRT-PCR	Ct	Visual Detection		SEM Detection	
		Sensitivity	Specificity	Sensitivity	Specificity
Positive (n = 30)	10.0 < Ct ≤ 15.0	100% (1/1)		100% (1/1)	
	15.0 < Ct ≤ 20.0	100% (1/1)		100% (1/1)	
	20.0 < Ct ≤ 25.0	87.5% (7/8)		100% (8/8)	
	25.0 < Ct ≤ 30.0	80% (4/5)		100% (5/5)	
	30.0 < Ct ≤ 35.0	0% (0/8)		87.5% (7/8)	
	35.0 < Ct ≤ 40.0	0% (0/7)		57.1% (4/7)	
	10.0 < Ct ≤ 40.0	43.3% (13/30)		86.7% (26/30)	
Negative (n = 15)	Ct > 40		100% (15/15)		93.3% (14/15)

Abbreviations: Ct, cycle threshold; qRT-PCR, real-time reverse transcription-polymerase chain reaction; SEM, scanning electron microscopy.

**Table 3:** SEM diagnosis with qRT-PCR and visual detection using LFA.

		Visual Detection ^a^			SEM Detection ^a^		
		Positive	Negative	Row Marginal	Positive	Negative	Row Marginal
SARS-CoV-2 qRT-PCR	Positive	13	17	30	26	4	30
	Negative	0	15	15	1	14	15
Column Marginal		13	32	45	28	18	45
Agreement (kappa Coef. ^b^)			0.622 (13 + 15)/45			0.889 (26 + 14)/45	

Abbreviations: LFA, lateral flow assay; qRT-PCR, real-time reverse transcription–polymerase chain reaction; SEM, scanning electron microscopy. ^a^ Number of samples in each setting are indicated. ^b^ kappa coefficient: the extent of agreement between frequencies of two sets of data collected on two different occasions.

## Data Availability

The raw data supporting the conclusions of this study will be made available by the authors without undue reservation.

## Supplementary Materials

The following supporting information can be downloaded at: https://www.mdpi.com/article/10.3390/biomedicines10020447/s1, Table S1. Comparison of densitometry data (mABS) with the dilution assay of SARS-CoV-2 nucleocapsid protein. TL: test line; Table S2. Comparison of particle counting with the dilution assay for SARS-CoV-2 nucleocapsid protein. BA: background area; TL: test line; Table S3. Comparison among visual, SEM, and qRT-PCR diagnoses of the nasopharynx, nasal cavity, and saliva samples. BA: background area; TL: test line
